# Exploring the potential role of telesurgery in the Middle East: insights from the recent Doha-Shanghai wet lab telesurgical connection

**DOI:** 10.5339/qmj.2025.32

**Published:** 2025-05-09

**Authors:** Ahmed Gamal, Abdel Rahman Jaber, Omar Aboumarzouk, Abdulla Al-Ansari

**Affiliations:** 1 Hamad Medical Corporation, Doha, Qatar; 2 AdventHealth Global Robotics Institute, Celebration, FL, USA *Email: Dr.Ahmedgamal88@gmail.com

**Keywords:** Telesurgery, remote surgery, robotic surgery, Qatar, China

Telesurgery is not a new concept; it began more than two decades ago with Jacques Marescaux’s groundbreaking Lindbergh Operation. This landmark surgery marked the start of a new era in remote surgical procedures. Using an undersea transatlantic cable, a surgeon in New York, USA, operated on a patient in Strasbourg, France, to perform a cholecystectomy.^1^ While the operation showcased incredible technological and cross-border collaboration, requiring extensive coordination between engineering teams and government entities, it came with significant challenges. Although it sparked optimism for the future of telesurgery, the following decades saw little progress in this field. Numerous obstacles, including prohibitive costs, limited connectivity, regulatory complexities, and an underdeveloped robotic and telecommunication infrastructure, hindered the widespread adoption and advancement of telesurgery technology at the time.^2,3^ Recent advancements in robotic technology and telecommunication connectivity have reignited interest in telesurgery, highlighting its potential to address disparities in healthcare systems.

The availability of faster and more reliable connectivity through Wi-Fi, 5G, and fiber networks, coupled with more cost-effective and remote-compatible surgical robots, has made telesurgery increasingly feasible.^4,5^ These innovations offer a promising solution to bridge the gap in healthcare access by delivering advanced medical services to underserved regions and enhancing patient accessibility to specialized care. Moreover, telesurgery holds significant potential for training and teleproctoring medical professionals, further advancing global healthcare equity and capacity.^6^

Robotic surgery and technology have gained significant interest and adoption in the Middle East, with multiple robotic surgical platforms now available and a notable increase in robotic surgery cases over the past few years. However, several challenges remain, one of such is the limited availability of robotic systems, which are still unavailable in many parts of the region. Additionally, there is a shortage of trained surgeons who are capable of performing robotic procedures, further hindering the widespread implementation of this advanced surgical approach.^7^

Building on the above, telesurgery technology has the potential to revolutionize healthcare in the Middle East by addressing these challenges. It can expand access to advanced healthcare services, bridging gaps in system availability and providing coverage to underserved areas. Additionally, telesurgery can play a pivotal role in training the younger generation of surgeons, enabling them to adopt and master robotic surgical techniques, thereby fostering the growth and sustainability of robotic surgery in the region.

Over the past few years, telesurgery has become a reality in China, driven by advancements in robotic platforms and telecommunication infrastructure. Multiple robotic platform companies in China now offer telesurgery capabilities, and numerous successful clinical trials have been conducted using these platforms, both within China and internationally.^5,8^ In this context, our team collaborated with MicroPort^®^ MedBot™, utilizing their Toumai platform to establish a telesurgical connection between Shanghai and Doha. This collaboration aimed to demonstrate the feasibility of a successful telesurgical connection while overcoming challenges related to connectivity and geographical distance.

On November 23, 2024, during the 21st Annual Arab Association of Urology Congress held in Doha, a surgeon from Qatar conducted a groundbreaking telesurgery experience, establishing a real-time connection between Itqan Clinical Simulation and Innovation Center in Doha and MedBot^®^ Mobile Showing & Training Center in Shanghai, China. The procedure involved a robotic-assisted remote simulated surgery performed on ex vivo tissue (a pig’s kidney). Our experiment adhered to China’s institutional animal care regulations and was approved by the Experimental Animal Ethics Committee, Shanghai (Approval No. 2024-001-098). This initiative involved overcoming significant distance and connection challenges. The direct round-trip distance was approximately 15,200 km, with a relay server established in Hong Kong to facilitate communication between the two locations. The surgeon console in Doha utilized a 4G network to connect to the relay server, experiencing a round-trip delay of approximately 140 ms. On the robot side in Shanghai, a broadband connection was used, with a round-trip delay of around 40 ms. Details of the connection can be seen in Figures 1 and 2. Latency remained within an acceptable range, allowing the surgeon to operate smoothly and efficiently. During the live demonstration, attendees observed that the surgeon’s movements in Doha were perfectly synchronized with the tissue manipulations performed by the robotic system in Shanghai. This setup successfully demonstrated the feasibility of conducting telesurgery over long distances, paving the way for future advancements in remote surgical procedures. While telesurgery applications offer groundbreaking potential, significant limitations remain, including high infrastructure costs, the need for safe and reliable connections, and complex regulatory and ethical considerations. Additionally, the purchase and maintenance of such robotic systems require substantial funding. Furthermore, technical expertise is still required at the patient’s location to ensure a safe and smooth process. Despite these limitations, the continued development and refinement of this technology can effectively bridge the gap between patients and advanced medical care, making complex surgical procedures and critical interventions more accessible to those who need them most, regardless of their location.

## Statements and declarations

The authors declare that no funds, grants, or other support were received during the preparation of this manuscript. The authors have no relevant financial or nonfinancial interests to disclose.

## Conflicts of interest

None.

## Figures and Tables

**Figure 1:**
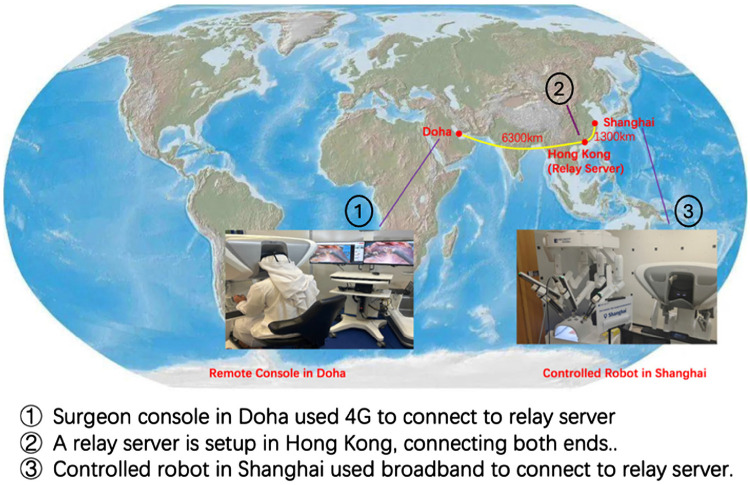
Network communication method: using a relay server in Hong Kong to facilitate communication between the surgeon console in Doha and the robot in Shanghai.

**Figure 2:**
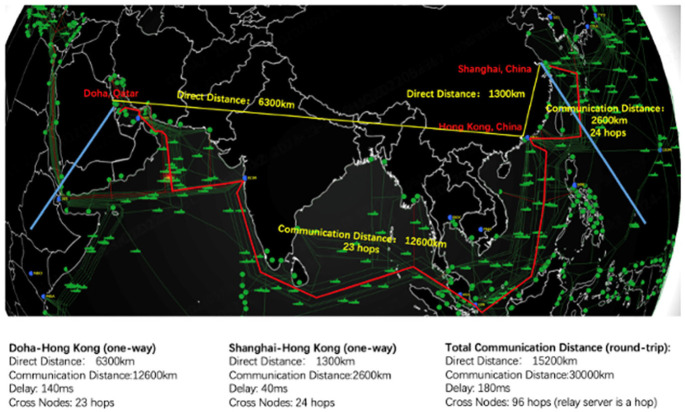
Network communication path analysis: showing communication distance (km) and signal delay (ms).
